# Exploring spirituality in palliative care services: an All-Ireland survey

**DOI:** 10.1186/s12904-025-01964-3

**Published:** 2026-01-05

**Authors:** Barry Quinn, John Wonnacott, Nipuna Thamanam, Niall Galligan, Lisa Graham-Wisener, Fiona Somers, Deirdre Murphy, Rory Cousins, Bríd McCarthy

**Affiliations:** 1 Queen’s University Belfast, Belfast, Northern Ireland UK; 2 Northern Health and Social Care Trust, Antrim, Northern Ireland UK; 3 University College Dublin, Dublin, Ireland; 4 Our Lady’s Hospice and Care Services, Dublin, Ireland

**Keywords:** Spirituality, Existential, Palliative Care, Meaning, Care

## Abstract

**Background:**

Spirituality and spiritual care are recognised as integral components of palliative care practice. Because of the diverse nature of this unique part of humanity, it may be challenging to describe what spirituality is or to ensure that spiritual care is delivered consistently in palliative care settings. However, failure to address the spiritual needs of a person and those who are important to them, who are in receipt of palliative care, can contribute to unnecessary distress.

**Methodology:**

This study employed a mixed-method design. Using a purposefully selected non-probability sampling method, an adapted survey was specifically developed and conducted across the island of Ireland among healthcare professionals working in palliative and end-of-life care services. Data analysis included the use of a computer software programme (IBM SPSS Statistics, Version 28), qualitative data was analysed using Braun and Clarke’s (2022) six step approach to thematic analysis.

**Results:**

Completed surveys were received from a wide range of health care professionals (152 responses). Results showed that 113 (74.8%) had been working in palliative care for at least 6 years, and 109 (72.1%) respondents reported frequent/very frequent engagement in this aspect of care. Four themes emerged from the qualitative data relating to the concept of spiritual care (1) linked to holistic care which was seen as fundamental to palliative care, (2) closely linked to supporting someone in their search for meaning, which was often associated with existential issues, (3) it required practitioners to recognise that people often expressed their spirituality and spiritual needs within the context of the individual’s values & beliefs, (4) a form of accompaniment, closely related to a journey that included supporting someone as they moved towards death.

**Conclusion:**

The findings from this study show that many members of the palliative care team are actively engaged in supporting people with life-limiting illness and their families with their spiritual needs and concerns. Respondents were able to offer a rich insight into what they believed spiritual care is and the important role it plays in delivering palliative care. There was a clear recognition of the need for further support and training. It is hoped that the findings from this study will contribute to further discussion, learning and research, and encourage more members of the palliative care team to engage in this component of person-centred care.

**Supplementary Information:**

The online version contains supplementary material available at 10.1186/s12904-025-01964-3.

## Background

Spiritual care is recognised as an essential component of palliative care practice and should be considered when a person and those who are important to them are facing a terminal illness [1]. As people approach the end of life, many confront existential questions about meaning, purpose, suffering and legacy [2]. These concerns may include religion, but also go beyond religious affiliation and delve into the core of what it means to be human [3]. Addressing spiritual needs with compassion is critical to improving the quality of life for any person and those who are important to them, who are the recipients of palliative care [4]. To date, research on this topic is sparse, with studies often focusing on theoretical developments rather than on data that can be used in clinical practice [5]. Promoting research and assessing current practices in the provision of spiritual care is therefore important.

Due to the wide, rich and diverse nature of spirituality as a concept, a consensus remains lacking on a singular definition of what spirituality actually is. According to deBrito Sena et al.. (2021) [6], whose systematic review sought to investigate its definitions in healthcare, their proposed spirituality framework outlined spirituality as a unique human attribute that is communicated through beliefs or practices to seek a connection with a source of meaning and self-growth. Despite previous claims by Sessanna, Finnell, and Jezewski (2007) [7] that spirituality and religion are mutual concepts, contemporary literature distinguishes them as being different [8, 9]. Moreover, spirituality is unique to each individual [10]. It encompasses a person’s search for meaning, connection, peace and transcendence [11]. Whilst it is acknowledged that some find this through traditional religious practices, others may turn to nature, relationships, art or personal values [6]. These ultimately result in the development of values, personal growth and better inner well-being [8]. An understanding of spirituality, without imposing assumptions or beliefs, is therefore important in healthcare for the provision of good spiritual care [6, 10].

Failure to address the spiritual needs of a person and those who are important to them, who are in receipt of palliative care, can contribute to unnecessary holistic distress [12]. Spiritual distress is a profound and often overlooked form of suffering that can arise when a person experiences a disruption in their sense of meaning or connection [10]. It can manifest as feelings of hopelessness, loss of meaning, guilt, or a sense of isolation from faith communities or significant others [13]. For some, it can be struggling with the idea of mortality or a feeling of abandonment [14]. The role of professional healthcare workers is to create a space if a person wishes to express, explore or find meaning in their experiences [15]. It can be done through meaningful communication. This involves active listening without judgement, asking open ended respectful questions, validating feelings and acknowledging the person’s struggle and finally supporting rituals, practices or connections that bring comfort [16]. Ultimately, the patient and those who are important to them, are central to the spiritual process and their voices, values and beliefs should guide the approach to care [6, 10]. Enabling patient and family to express their spiritual needs and participate in the care decisions honours their dignity and autonomy. Identifying and addressing spiritual needs can therefore alleviate and avoid spiritual distress.

In palliative care, the responsibility of providing spiritual care does not rest on a single individual or with only some members of the health and social care team. It is a shared commitment of the entire disciplinary team [15]. While chaplains or pastoral care workers are uniquely trained to provide in-depth support, every member of the team plays a vital role in recognising and responding to spiritual concerns [17]. Moreover, the work of chaplains and pastoral workers is enhanced when integrated into a collaborative care model where they are informed by and inform the team’s understanding of a patient’s overall well-being [17]. As patients and their significant others face the profound realities of life and death, spiritual care can provide comfort, healing, and a sense of peace [16]. Through the provision of good spiritual care that is culturally and ethically sensitive, healthcare professionals can offer truly holistic care that honours the whole person, body, mind, and spirit [18]. It is therefore critical that all healthcare professionals are both comfortable and competent in the delivery of spiritual care in the palliative care setting. However, there is a limitation regarding the lack of adequately trained healthcare professionals in this aspect of care [10, 12]. This study focuses on current practice and need in relation to spiritual practices in palliative care.

A special interest group within the All-Ireland Institute of Hospice and Palliative Care (AIIHPC) was established in June 2023. The group aims to provide a collaborative multi-disciplinary space for members to work on identifying priorities in better understanding and delivering psychological, social, and spiritual care in palliative and end-of-life care settings. By researching psychological, social, and spiritual care, it enables the group to generate the evidence needed to change practice.

This survey aimed to explore current practices and needs in responding to and delivering spiritual support within palliative and end-of-life care services across the island of Ireland. This would help support in identifying and developing research study ideas that would address current spiritual needs, and in developing collaborative research funding proposals in order to secure resources to address these identified priorities.

## Methodology

### Study aim

To explore the role of spiritual care within palliative and end-of-life care services across the island of Ireland.

### Study objectives

1. To examine current practice in delivering spiritual care in palliative and end-of-life care services.
2. To identify training needs to support the delivery of spiritual care in palliative and end-of-life care services.
3. To help prioritise and guide research and training in supporting the delivery of spiritual care in palliative and end-of-life care services.

### Design and setting

This study employed a mixed-method design and was conducted across the island of Ireland.

### Sample and population

The setting for the study is the island of Ireland, including Northern Ireland with a population circa 1.9 million [19] and the Republic of Ireland with a population circa 5.3 million [20]. The eligibility criteria for the study included all healthcare professionals (>18 years) working in palliative and end-of-life care services across various settings. The participants were drawn from within and beyond the AIIHPC membership; members were invited to share the survey with their peers. A purposefully selected non-probability sampling method was employed. Eligibility criteria included those working in roles that directly supported patients and/or significant others as part of a palliative and end-of-life care service in Ireland.

### Tool development

An adapted survey instrument was specifically designed to explore current practice and need in spiritual support within palliative and end-of-life care services across the island of Ireland. A review of the literature showed that no previously used surveys were available that specifically addressed the focus of the study. Using the work of Vivat et al. (2023) [21] and Eva and Morgan (2018) [22] the adapted survey was created by the research team, including feedback from external experts, users and carers. Following this, the research team adapted or refined the questions. To assess the understandability of the survey, two independent experts reviewed and refined the questions. Final refinements of the survey were done after piloting on a small group of people. The final survey can be found in appendix 1.

### Data collection method

Healthcare workers comprised doctors, nurses, social and pastoral care workers, chaplains, allied health professionals and psychologists working in palliative care across the island of Ireland were invited to participate in the study by the AIIHPC. The participants were drawn from within and beyond the AIIHPC membership. AIIHPC members were also invited to share the survey link with healthcare workers working in palliative care. An online survey, created using MS Forms, was used to collect data. The survey and an information sheet were sent out via email and social media by the AIIHPC to all its members. AIIHPC members were encouraged to complete and also share the survey link with colleagues who might wish to complete the survey. AIIHPC members received two reminder messages. The first reminder message via email was sent after two weeks, and a final reminder was sent one week prior to the survey’s closure. The email link to the survey included a brief invitation letter and a participant information sheet, which explained the purpose of the study and the inclusion criteria. At the beginning of the online survey, the participants were asked to tick a consent box before continuing with the survey. Participants were informed that all responses would be anonymous. The survey consisted of five parts: demographics, introductory questions, questions on spiritual care and training needs.

### Data analysis

The survey data in the current study were analysed using a computer software programme, the IBM SPSS Statistics, Version 28. All the questions were cross-checked for errors and completeness before data entry. Descriptive data analysis of each survey item was conducted using descriptive statistics. Descriptive statistics were used to describe the sample’s demographic characteristics. The qualitative component of the survey was analysed using Braun and Clarke’s (2022) [23] thematic analysis to identify patterns and themes within the data. The analysis followed a six-step process involving familiarisation with the data, generating initial codes, searching for themes, reviewing themes, defining and naming themes, and writing the report.

### Ethical considerations

The study was reviewed and approved by the Medical and Health Life Science Ethics Committee at Queen’s University Belfast. Participants were required to sign a consent form prior to commencing the survey. The participants were informed that their participation was voluntary and that they had the right to refuse to participate or withdraw from the survey at any time prior to completion of the survey. The privacy and confidentiality of the study participants were ensured, and all data from the surveys were stored electronically on an encrypted, secure server to which only members of the research team had access. The data was managed in line with the research ethics committee’s guidelines. As the survey was completed anonymously, participants were informed that once they submitted the survey online, it was not possible to remove their data from the study thereafter.

## Results

One hundred fifty-two people (152) responded to the survey, of whom 138 (91%) were aged 35 years or older (Table 1). Results showed that 113 (74%) had been working in palliative care for at least six years; of these, 88 (58%) had worked in this area of care for over 10 years (Table 1). Respondents worked in a variety of settings where palliative care was delivered, including hospice 56 (37%), community 45 (30%), hospital 25 (17%), and 19 (12%) worked in other settings. The remaining respondents reported that they worked across all three settings 6 (4%). The highest number of respondents were nurses 69 (46%), followed by social workers 18 (12%), chaplain/pastoral workers 14 (9%), occupational therapists 10 (7%) doctors 8 (6%), and the remaining 30 (20%) respondents worked as a physiotherapist (5), dietician (5), pharmacist (4) or psychologist (3) and other (13), three respondents did not respond to this question. Respondents were asked whether they had a religious affiliation or were associated with any spiritual beliefs. Of the 151 participants who responded to this question, the majority of respondents identified as being affiliated with Christianity, at 107 (70%). Twenty-eight (18%) respondents described themselves as spiritual but not religious, and 12 (8%) described themselves as neither spiritual nor religious. The remaining 4 respondents identified as Agnostic, Buddhist or Humanist and non-practising Catholic (4%) (Table 1).

**Table 1:** Demographics

Questions	Response Choices	No of participants	Valid %	Total
Age	18–24	1	1%	152
	25–34	13	9%	
	35–44	34	22%	
	45–54	55	36%	
	55–64	47	31%	
	65–74	2	1%	
How long have you been engaged in supporting people receiving palliative and end-of-life care?	Less than a year 1–5 yrs 6–10 yrs > 10 yrs	8 31 25 88	5% 20% 16% 58%	152
Religion/Spirituality	Christianity	107	70%	151
	Spiritual but not religious	28	18%	
	Neither spiritual nor religious	12	8%	
	Agnostic	1	1%	
	Buddhism	1	1%	
	Humanism	1	1%	
	Non-Practising Catholic	1	1%	

Respondents were invited to use free text to describe the role of spiritual care support in palliative care. All of the respondents answered this question, leading to a rich variety of responses which, when reviewed and analysed by the research team, resulted in four key themes (Table 2).

**Table 2:** Spiritual Care Support

Theme 1	Spiritual care is closely linked with holistic care, which is fundamental to palliative care
Theme 2	Spiritual care is closely linked with an individual’s search for meaning
Theme 3	Spirituality and spiritual needs are linked to an individual’s values and beliefs
Theme 4	Spiritual care as a form of accompaniment

The first theme focused on spiritual care being linked to the concept of holistic care which was seen as fundamental to palliative care. As one respondent described, ‘*Spiritual care is an essential component of offering holistic care’.* This care included the person living with advanced disease and those who are important to them. Spiritual care was perceived as having a contribution to make in symptom management of the child or adult living with advanced disease, which included engaging with the concerns of family members. *‘Spiritual pain contributes to the overall ‘total’ pain experience that our children and families experience and it’s essential that professionals know how to explore needs and offer the right support’.* To fully address the concept of total pain, the team needed to have some understanding of what spiritual pain was, in order to offer support and manage pain and other symptoms.

The second theme was that spiritual care was closely linked to supporting someone in their search for meaning, which was often associated with existential issues. This was expressed by one respondent in the following statement. *‘Taking into consideration*,* in essence*,* the whole person - giving space to finding meaning in death and dying’.* Through ‘giving space’, to spiritual care, the person might find meaning in and through what they were experiencing, their own death and dying. Spirituality was not seen as simply part of what made a person, but was central to the notion of personhood, central to the palliative care approach.

The third theme recognised that spiritual care required practitioners to recognise that people often expressed their spirituality and spiritual needs within the context of the individual’s values and beliefs. This was described by one respondent in the following way. *‘Spiritual care relates to attending to and responding to the beliefs and values of the person living with advanced illness.’* Respondents recognised that these values and beliefs may or may not be expressed through a formal religion.

The final theme developed was that of spiritual care as a form of accompaniment, closely related to a journey that included supporting someone as they moved towards death. As one respondent described it, *‘It is walking the palliative care journey alongside them’.* The idea of walking with, being alongside the person (patient, significant others, carer), was seen as a key part of the respondents’ role in delivering spiritual care.

Having described their own idea of what spiritual care was, respondents were asked how often they were involved in assessing and supporting patients’ and significant others/carers’ spiritual needs. One hundred and nine respondents (72%) recognised their engagement in this aspect of care, of which 50 stated they were engaged very frequently, and 59 stating they were involved frequently, in assessing and supporting people’s spiritual care needs. Forty three respondents (28%) stated that they were rarely or never involved in this aspect of care (Table 3). One hundred and thirty-three respondents stated that they had rarely or never received any training focused on spiritual care. However, 134 (88%) respondents stated they were fairly competent, competent or very competent in supporting patients’ and significant others/carers’ spiritual needs. Eighteen respondents (12%) stated that they lacked competence in this aspect of care (Table 4).

**Table 3:** How often are you involved in assessing and supporting patients and family carers’ spiritual needs

Response Choices	No of participants	Valid %	Total
Very Frequent	50	33%	152
Frequent	59	39%	
Rarely	32	21%	
Never	11	7%	

**Table 4:** How would you rate your competence in supporting patients’ and family/carers’ spiritual needs?

Response Choices	No of participants	Valid %	Total
Very Competent	27	18%	152
Competent	57	37%	
Fairly Competent	50	33%	
Not Competent	18	12%	

The survey asked respondents how they assessed patients and significant others/carers spiritual needs, and they were given a number of options from which respondents could choose multiple answers. The highest number of respondents selected talking with the patient (82%) and talking with the family (76%). Respondents also referred to observing patients (41%) and reviewing the relevant medical/nursing notes (34%). For many respondents, their initial assessment led them to request a review by a member of the chaplaincy and pastoral care team (36%) (Table 5).

**Table 5:** How do you assess patients’ and family/carers’ spiritual needs?

Response options	No of Participants	Valid %	Total
Talking with the patient	125	82%	152
Talking with family	116	76%	152
Observing the patient’s presentation	62	41%	152
Arranging a review with a chaplain/pastoral care worker	55	34%	152
Through a patient’s medical/nursing records	51	36%	152
Other	31	20%	152

Following the identification of spiritual care, respondents were asked to describe how support was offered; the respondents could choose multiple answers. Of the total respondents (150), 102 (68%) respondents spoke of offering themselves as a form of support, 87 (58%) spoke of referring the person to the pastoral care team, and referring the person to chaplain/pastoral care worker externally 40 (26.6%). Respondents described making a referral to the psychology or counselling team 38 (25.3%), referring to an external support service 21 (14%), referring to a psychologist or counsellor externally 16 (11%) (Table 6).

**Table 6:** If you identify any type of spiritual needs in a patient or a family/carer, what do you do next?

Response options	No of Participants	Valid %	Total
Offer them my support	102	68%	150
Refer to the chaplain/pastoral care worker on the team	87	58%	150
Refer to a chaplain/pastoral care worker externally	40	26.6%	150
Refer to a psychologist or counsellor on the team	38	25.3%	150
Refer to an external support service	21	14%	150
Refer to a psychologist or counsellor externally	16	11%	150

The support offered to patients and significant others/carers also included giving the person an opportunity to talk 139 (92%), offering to bring the person to a quiet place 69 (45.6%) or garden 48 (31.7%).

Although the respondents were engaged in assessing and delivering spiritual care, clear barriers were identified. 44% of respondents identified the limited access to spiritual care support/practitioners, and 64 (42.4%) of respondents identified the lack of time to address this aspect of care. Another perceived barrier related to the belief that some patients, significant others, and carers were unwilling to talk about their spiritual concerns 51 (33.7%).

Finally, respondents were asked what further training would help them in being able to identify and support patients’ and significant others/carers’ with spiritual care needs (Appendix 1). Respondents wanted help in further understanding the notion of spirituality and spiritual care, which included practical guidance on how to identify, assess and address these needs. There was a recognition of the need for a better understanding of the rich diversity of need, related to this aspect of care. This included a greater awareness of the many religions and cultures that exist, and a greater sensitivity to those who might feel excluded or ostracised from spiritual care support.

## Discussion

The majority of respondents who had a broad range of experience working in the field of palliative care reflected the global view that recognises the role of the person with a life-threatening condition, the significant others members and caregivers, and the central role of spirituality in this aspect of clinical practice [24, 25]. In this current study, responding to spiritual needs and the delivery of spiritual care was not perceived as the domain of any one profession; instead the majority of respondents, representing a broad range of professions, were engaged in this important aspect of care. This reflects the literature that a wide spectrum of health and social care professionals are actively engaged in delivering spiritual care [26, 27]. Miller et al., (2023) [27] perceive spiritual care not simply as another item for a nurse to address but a core component of everyday practice, and Gijsberts (2022) [28], who focused on the importance of doctors engaging with this aspect of care. Additionally, Austin et al.’s (2025) [29] review showed the benefits that spiritual care brings when all of the team are engaged.

Interestingly, assessments in this aspect of care were often described by respondents as a conversation, a chat or simply observing the person, rather than a formalised assessment approach. No respondent referred to a spiritual assessment tool however, respondents did ask for support in being better able to assess people’s spiritual needs. Quinn (2018) [30] talks about the importance of story-telling among those approaching the end of life, and the need for that story to be heard by a caring practitioner. Respondents gave clear examples of the spiritual care they offered, including offering support, giving the person time and space, allowing them to talk about what concerned them, offering to take the person to a quiet place, a garden or a prayer room, or offering the support of another experienced member of the team. Recent studies have shown similar practical approaches to delivering spiritual support in palliative care [27, 29].

Alongside this reality, there was a recognition that while respondents recognised and valued their role in this aspect of care, they also valued the role and the support of chaplains, pastoral care workers, psychology and counselling staff, who they appeared to work closely with. This close working relationship was observed when a spiritual need was identified, one of the possible actions was to signpost to a chaplain, pastoral worker or a member of the psychology and counselling team. While the role of the chaplain or psychologist is clearly different, each of these roles was perceived by many respondents as key in supporting the person with their spiritual needs and that these members of the team had skills and experience which could further support the person in need.

The majority of respondents identified with some form of belief or value system. For many of the respondents their belief system was linked to Christianity. This was no surprise as a recent national survey on religious beliefs in Ireland showed that over 71% identified as Christian [31]. However, the same census recognised that the number of people with no religious affiliation was continuing to rise, reflecting the change of beliefs and practices within Ireland. Similar to other studies [8], spiritual care was seen as engaging with the person’s values and beliefs, which may or may not fit within a religious concept. There was an acknowledgement that whether or not a person had a religious faith, each person had values and beliefs that were important to them. This reflects the definition of spirituality offered by the European Association of Palliative Care that recognises spirituality as an important part of human life which is linked to the way a person or a group of people experience and/or seek meaning and purpose in life [22]. The participants’ responses regarding their ideas of what spiritual care involves, demonstrates a rich insight into how they believed spiritual care was delivered in palliative care services. The respondents saw a clear connection between the aims of palliative care and the need for spiritual care which is similarly reflected in the global view of what palliative care is [24, 25, 32].

Similar to other research, many of the participants in this study described spiritual care as supporting people in their search for meaning, particularly in the light of the person’s advancing condition. Frankl’s (1997) [33] seminal work acknowledges that this search for meaning is a core component of every human being which becomes more visible at key life events including approaching one’s death. In a more recent study, Quinn (2018) [30] showed that the search for meaning was an important component of people’s experience of living with cancer, including advancing disease. In Quinn’s (2018) study [30], many of the people interviewed acknowledged that they were moving towards the end of their life, and while not everyone found meaning in their experiences, the process of searching, was important, alongside the support offered by caring practitioners. This search process and the need for support was reflected in the current study in the respondents’ description of spiritual care. The respondents recognised that alongside their vital role in supporting people living with advanced disease and conditions they were also supporting significant others who were also engaged in this search for meaning process. This process required each of the respondents to journey with the person or to accompany the person at an uncertain and for many, a difficult time in their life as they faced the reality of death. Perhaps this idea of accompaniment is also reflected in the importance of conversations and interacting with patients and significant others as described by the respondents in the study.

The current study acknowledged that in order to fully address symptom management, including pain, the spiritual component of that symptom or that person needed to be recognised and addressed. Miller et al. (2023) [27] also acknowledged that spiritual care has an important role to play in the management of pain and other symptoms alluding to a holistic approach to symptoms which the respondents in this study recognised. Dame Cicely Saunders vividly described the concept of total pain including suffering, as one that requires the health care professional to fully engage with the dying person [34]. Quinn et al. (2017) [35] suggested that in order to move beyond the biophysical approach to pain or indeed any symptom, each symptom needs to be seen as a disruption or a disturbance in the relationship the person has with their body, emotions, beliefs and values, and their place in the world.

Although there was a clear and articulate expression of the spiritual care which the majority of the respondents were engaged in, there was also a plea for more training education and support on aspects of spiritual care. The need for training and education went beyond simply providing teaching sessions and included a request for practical guidance and concrete support. This included a need to have an even deeper understanding of what spiritualty and religion actually meant and how it might be expressed within the palliative care setting. Puchalski et al. (2020) in developing training for those working in palliative care focused on supporting all clinicians in being able to provide generalist spiritual care, with trained chaplains and pastoral workers being able to provide specialist spiritual care [36]. While focusing on the need for training in this area of care it is important to recognise the reality of the person in need, recognising the interconnection of the spiritual, psychological, social and physical reality of living with an advanced disease or condition [37]. Alongside this need for better understanding was a need to increase their own awareness of the different cultures of those they supported, perhaps reflecting the growing diversity across the island of Ireland. Linked to the developments seen within palliative care, and the growing diverse needs of people, including those they supported, respondents had a desire to support those who might feel excluded from some religions, religious practices or who might feel ostracised from their religion of birth. The need to recognise and respond to the diversity of cultural and spiritual need within palliative care is clearly supported by the European Association of Palliative Care [38]. While not everyone will identify or feel part of a religion, as Frankl (1997) [33] and others have stated [24, 30], being human necessitates a spiritual component and when faced with a terminal illness this might take on even more importance.

Perhaps the support and training that respondents requested, including a better understanding of spirituality and spiritual assessments could be offered in a variety of ways. While there may be a need for formal training sessions and workshops there may also be an opportunity for more experienced members of staff including nurses, chaplains and members of the psychology/counselling team to role model spiritual care delivery. For example, in the past some chaplains may have tended to work more independently when seeing the patients referred to them and the only engagement with staff might be to find out where a patient was [39]. Having chaplains participating in the multi-professional team meetings can be an opportunity for the chaplain to learn more about the concerns of patients and significant others and to support staff as they in turn engage with patients and significant others, and to be more aware of the challenges that staff face each day. Another suggestion of where role modelling can take place when it is deemed appropriate, can occur when a chaplain and a nurse carry out a joint visit to a person’s room or home. This joint visit can be an opportunity to reflect the engagement of all staff in this aspect of care and for the nurse and chaplain to learn from one another [40].

Interestingly, but not surprising, there was a recognition that in order to support people with their spiritual needs and concerns, those working in palliative care asked for guidance and support in taking steps to care for themselves. This perhaps is linked to the idea of accompaniment described by some respondents and were the respondent might also be engaged in their own soul searching as they supported people approaching death. This recognition perhaps reflects the reality of being faced with death and dying, and engaging in deeply personal conversations that could have a toll on the listener.

One of the key elements of being able to deliver spiritual care has been referred to as knowing oneself [27]. If one is to be present to supporting a person in their search for meaning, then the one who accompanies also needs to have some awareness of who they are and their own spirituality [41]. An awareness of one’s own spirituality and how it is being lived out in one’s life may place the person in a better position to participate in the role of being a companion to others. This reflects Campbell’s (1984) [42] idea of the ‘skilled companion’, whereby each health care professional uses both their clinical skills and their humanity to walk alongside people as they journey though challenging times in their lives. This self-knowledge and indeed self-care could be enhanced through reflective practice which is part of many palliative care services. The respondents recognised that self-care was vital in this work in order for each practitioner to keep engaging with this core aspect of who people are. The danger is that if practitioners become worn out, they may be less able to engage with this important component of care.

### Strengths and limitations

The mixed method study provides insight into the thoughts and needs of healthcare professionals working in the field of palliative care across the island of Ireland in relation to spirituality and spiritual care. It demonstrates that respondents engaging in this aspect of care had a clear idea of what spirituality is, how it might be expressed and how spiritual needs and care could be addressed and responded to. The study findings could be used to help promote learning and further discussion in the development of spiritual care in palliative services.

Although the study focused on health care professionals, it did not seek the thoughts of people living with advanced disease, family members/significant others and other health care workers. A further study is required to address this gap. The study focused on the island of Ireland, where many of the respondents identified as Christian; therefore, the findings may not be generalisable to other countries and settings.

## Conclusion

The need for spirituality to be recognised and more incorporated into palliative care practice continues. The findings from this mixed methods study show that many members of the palliative care team are actively engaged in supporting people with life-limiting illness and their families with their spiritual needs and concerns. Respondents were able to offer a rich insight into what they believed spirituality is and the vital role it plays in delivering palliative care. Despite a lack of training in this aspect of care, many respondents felt confident in engaging with this aspect of care, although there was also a clear recognition of the need for further support and training. It is hoped that the findings from this study will contribute to further discussion and learning, and encourage all members of the palliative care team to engage in this vital component of person-centred care.

## Supplementary Information

Supplementary Material 1.

## Data Availability

The data that support the findings of this study are available upon reasonable request from the first author (BQ). The data are not publicly available due to privacy and ethical restrictions.
